# Two-stage-method-based calculation and analysis of the deformation of the existing subway tunnel caused by the diagonal crossing of the new tunnel

**DOI:** 10.1038/s41598-026-40967-9

**Published:** 2026-02-28

**Authors:** Yonghui Li, Yucheng Zhao, Gang Shi, Xiaoming Wang, Shuai Wu, Wenmin Yao

**Affiliations:** 1 College of Civil Engineering, Zhengzhou University, Zhengzhou, Henan China; 2 State Key Laboratory of Tunnel Boring Machine and Intelligent Operations, Zhengzhou, Henan China; 3 China Construction Fifth Engineering Division Fourth Construction Co., Ltd., Luoyang, Henan China

**Keywords:** Shield tunnel, Pasternak foundation beam, Mindlin’s method, Theoretical calculation, Engineering, Mathematics and computing

## Abstract

This study presents a theoretical analysis of the deformation induced in an existing curved subway tunnel by a new shield tunnel crossing diagonally beneath it. A refined two-stage method is developed to address this engineering problem. In the first stage, the additional stress on the existing tunnel is calculated using Mindlin’s solution. In the second stage, the existing tunnel is modeled as an Euler–Bernoulli beam on a Pasternak foundation, explicitly incorporating the effects of tunnel curvature and a stress reduction factor for the grout-reinforced zone. The proposed method is validated against monitoring data from a case study of the Zhengzhou Metro, showing good agreement. A systematic parametric analysis investigates the influence of key factors: the clearance and intersection angle between tunnels, the curvature radius of the existing tunnel, the length of the grouted section, and Poisson’s ratio of the grouted soil. Results demonstrate that the crossing angle and grouting length are the most significant parameters affecting deformation, whereas the existing tunnel’s curvature and the grout’s Poisson’s ratio have a negligible impact.

## Introduction

Urbanization and population increase have resulted in extensive development of urban underground transportation. An increase in the number of transportation lines has resulted in new lines crossing existing lines, altering the terrain^1–3^ and affecting the stability and safety of surrounding buildings.

Many international and domestic scholars have conducted extensive research on the deformation of existing tunnels caused by new tunnel construction. A theoretical method cannot always provide accurate information on tunnel deformation. However, theoretical equations can be used to calculate the tunnel deformation and change in forces. The two-stage method has been commonly used to analyze the impact of new tunnel excavation on existing tunnels^4–6^.

The first stage of this method analyzes the additional stresses on the existing tunnel due to the new tunnel excavation. Mindlin et al.^7^ regarded the tunnel as a circular hole in a semi-infinite, homogeneous, isotropic, elastic medium affected by gravity only and analyzed the stress characteristics around the tunnel to derive an analytical solution. Sagaseta et al.^8^ assumed that the soil was an isotropic, elastic medium. Absolute displacement was used to determine the surface settlement due to new tunnel construction. Loganathan et al.^9^ calculated the strata loss rate following Sagaseta et al.^8^ and obtained an analytical solution for the vertical displacement of the soil around existing tunnels. Liu et al.^10^ considered the rotation and shear effects of the nodes between the rings of tubes and calculated the longitudinal deformation of existing tunnels based on the minimum potential energy. In the second stage, the existing tunnel is regarded as an infinite-length beam based on different foundation models. Differential equilibrium equations are established, and the vertical displacement of the existing tunnel is obtained by numerical solutions. Many domestic and international scholars have used the Winkler foundation, Pasternak’s two-parameter, and Kerr’s foundation models as the elastic foundation beam model. The Winkler foundation model has been used to determine the deformation of existing tunnels during overcross tunnel construction^11,12^.

The Kerr foundation model uses the c-value based on the Pasternak foundation model, providing a more realistic model. The parameters of the Kerr foundation model can be determined by comparing the results of a theoretical analysis and numerical simulation^13–15^, the c-value in the foundation model is difficult to determine, but three parameters provide more accurate results. However, the calculation is complex and unsuitable for engineering applications. The Winkler foundation model has only one parameter (the stiffness *k* of the spring) describing soil deformation, resulting in a large error. Therefore, the Pasternak model is typically used in practical applications. It reflects the shear and compressive stresses of the soil body, provides accurate results, and has low computational complexity^16,17^. Ding et al.^18^ calculated the deformation of existing tunnels caused by the crossing of a large-diameter mud-water shield in saturated soil using the two-stage method. They considered the construction factors, such as the additional force by the mud-water shield on the excavation surface, the friction of the shield shell, and the grouting pressure at the end of the shield.

Recent research has increasingly emphasized the importance of integrating specific geological and construction parameters into predictive frameworks. For instance, Lai et al.^19^ developed a performance prediction model for cut-and-cover tunnel boring machines, establishing quantitative relationships between rock mass classification indices and engineering responses, thereby highlighting the value of parameterizing key influencing factors. Concurrently, Zhao et al.‘s^20^ research on large deformation mechanisms in specific rock masses such as foliated basalt demonstrated how intrinsic geological structures govern deformation patterns, reflecting the scenario-dependent nature of tunnel behavior. Furthermore, advances in field testing methods for determining critical geotechnical parameters, such as the elastic modulus, underscore the foundational role of reliable input data in analytical models^21^. Collectively, these studies point toward an approach that combines mechanistic modeling with well-characterized site-specific conditions. In the context of shield tunneling beneath existing infrastructure, this implies that a robust deformation prediction method should not only be grounded in sound mechanics but also capable of incorporating distinctive project features, such as the alignment curvature of the existing tunnel and the reinforcement effects of mitigation measures like grouting.

However, many theoretical studies have not considered curved tunnels or the influence of the existing tunnel wall behind the ring grouting reinforcement. Recent analytical studies have underscored the importance of incorporating complex practical factors into theoretical frameworks. For instance, this involves systematically considering the sequential installation of supporting components and developing viscoelastic solutions. These advances highlight a trend toward enhancing the physical realism of analytical models. Therefore, this paper conducts a theoretical analysis of the deformation of existing tunnels. A two-stage method is used to simplify the theoretical model of the soil body based on Mindlin’s and Pasternak’s foundation models. The radius of curvature of the existing curved tunnel and the stress reduction of the grout-reinforced section are considered. A computational model of the deformation of the existing curved tunnel crossed diagonally by a shield tunnel is established, and the deformation derived from the theoretical analysis and a numerical simulation is compared. The deformation of the shield tunnel is analyzed under different crossing conditions. The effects of the distance and angle between the old and new tunnels, the curvature radius of the existing tunnel, the length of the grouting section, and Poisson’s ratio of the grouted area on the deformation of the existing tunnels are assessed.

## Calculation method and models

### Computational models and assumptions

Since the construction of the new tunnel causes soil unloading and additional stress, the existing tunnel will move upward. Therefore, the following assumptions are made:

1. The existing tunnel is a semi-infinite elastic space, and the model contains homogeneous and isotropic terms.
2. Only the soil unloading caused by the excavation of the new tunnel is considered.
3. The redistribution of the soil stress occurs instantaneously.
4. The existing tunnel is modeled as Euler-Bernoulli beams based on the Pasternak model.
5. The soil consolidation and creep are not considered.

**Fig. 1:**
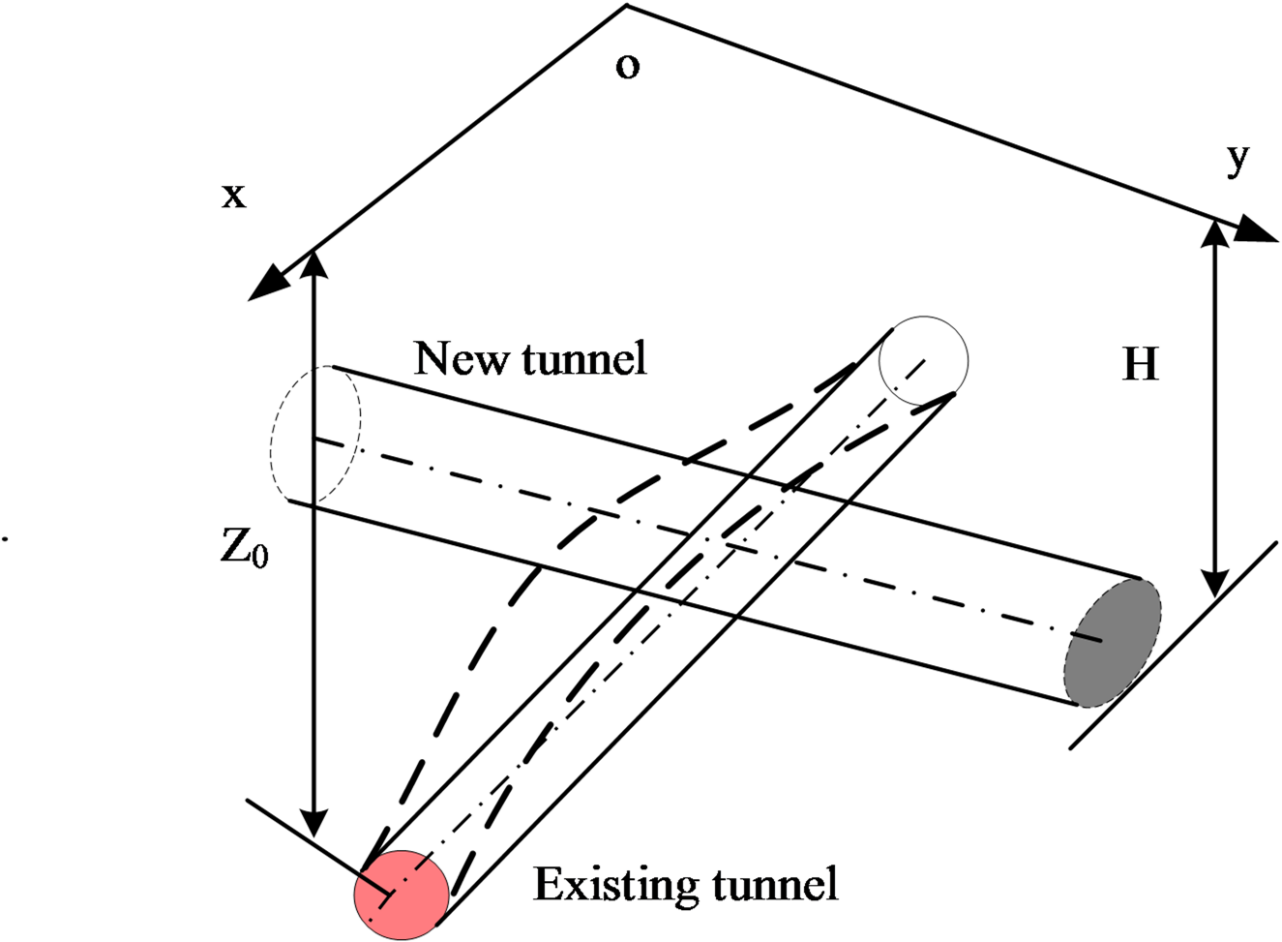
Calculation model.

The computational model is shown in Fig. 1.

### Additional stress caused by the new diagonally crossing tunnel

The load released by tunnel excavation per unit length is the weight of the excavated soil mass minus the weight of the newly installed segment:

1 $$q=\frac{{{\gamma _{\mathrm{s}}}\pi {R^2} - {\gamma _{\mathrm{t}}}\pi \left( {R_{2}^{2} - R_{1}^{2}} \right)}}{{2R}}$$

where *q* is the released load per unit length, ${{\mathrm{\boldsymbol{\upgamma}}}_{\mathrm{s}}}$ is the excavated soil weight, ${{\mathrm{\boldsymbol{\upgamma}}}_{\mathrm{t}}}$ is the weight of the shield segment, ${R_1}$ is the inner radius of the shield segment, ${R_2}$ is the outer radius of the shield segment, and *R* is the outer radius of the shield machine.

**Fig. 2:**
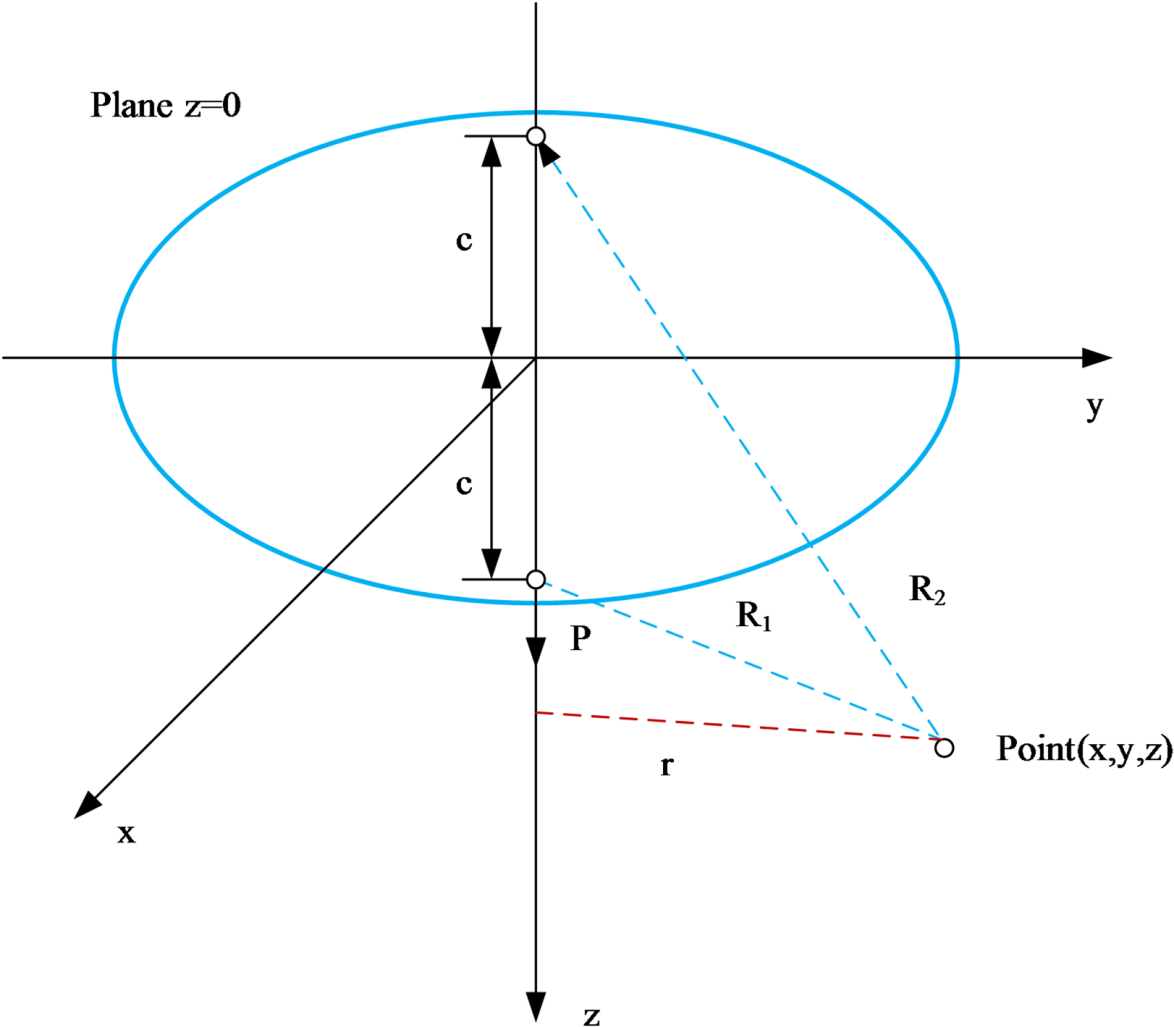
The model of Mindlin’s solution.

According to^7^ solution, when a vertical concentrated load *P* is applied at depth c to the soil, the additional vertical stress at any point (*x*,* y*,*z*) is expressed as follows Fig. 2.

2 $$\begin{gathered} {\sigma _z}=\frac{P}{{8\pi \left( {1 - \mu } \right)}}[ - \frac{{\left( {1 - 2\mu } \right)\left( {z - c} \right)}}{{R_{1}^{3}}}+\frac{{\left( {1 - 2\mu } \right)\left( {z - c} \right)}}{{R_{2}^{3}}} - \frac{{3{{\left( {z - c} \right)}^3}}}{{R_{1}^{5}}} \\ - \frac{{3\left( {3 - 4\mu } \right)z{{\left( {z+c} \right)}^2} - 3c\left( {z+c} \right)\left( {5z - c} \right)}}{{R_{2}^{5}}} - \frac{{30zc{{\left( {z+c} \right)}^3}}}{{R_{2}^{7}}}] \\ \end{gathered}$$

3 $${R_1}=\sqrt {{x^2}+{y^2}+{{\left( {z - c} \right)}^2}}, {R_2}=\sqrt {{x^2}+{y^2}+{{\left( {z+c} \right)}^2}}$$

where *λ = 2Gν/1−2ν​* is Lamé’s first parameter. Substituting the expressions for displacements *u*,* v*,*w* from Mindlin^7^ and performing the differentiation yields the full expression for *σzσz​.* For the case of a distributed tunnel excavation load *q* acting over a finite length, Eq. (2) in the main text is obtained by integrating the point-load solution along the tunnel axis, applying the principle of superposition, and relating the concentrated load *P* to the distributed load *q* per unit length through *P* = *qdy*. This integration accounts for the geometry of the diagonally crossing tunnel and leads to Eqs. (4)–(6) used in the two-stage analysis.

**Fig. 3:**
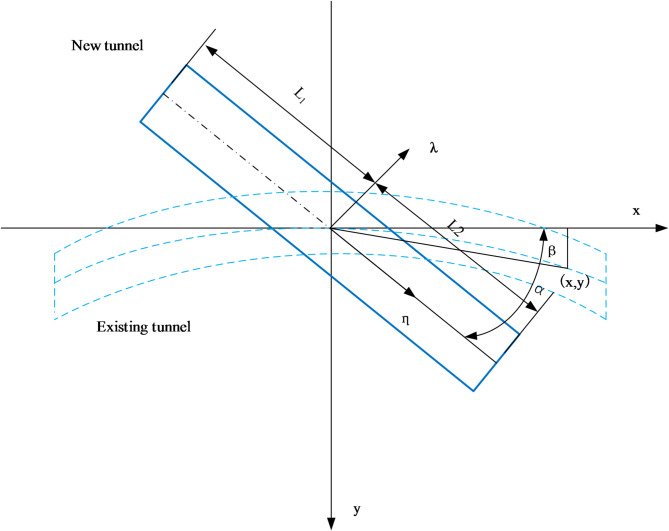
Location diagram of the new and existing tunnels.

Figure 3 indicates a bend in the existing tunnel with a curvature radius of *r.* The additional stress of the existing tunnel due to the influence of the new tunnel is expressed as:

4 $$\begin{gathered} \sigma \left( x \right)=\mathop \smallint \nolimits_{{ - {R_0}}}^{{{R_0}}} \mathop \smallint \nolimits_{{ - {L_1}}}^{{{L_2}}} \frac{{qd\lambda d\eta }}{{8\pi \left( {1 - \mu } \right)}}[ - \frac{{\left( {1 - 2\mu } \right)\left( {{Z_0} - h} \right)}}{{R_{1}^{3}}}+\frac{{\left( {1 - 2\mu } \right)\left( {{Z_0} - h} \right)}}{{R_{2}^{3}}} - \frac{{3{{\left( {{Z_0} - h} \right)}^3}}}{{R_{1}^{5}}} \\ - \frac{{3\left( {3 - 4\mu } \right){Z_0}{{\left( {{Z_0}+h} \right)}^2} - 3h\left( {{Z_0}+h} \right)\left( {5{Z_0} - h} \right)}}{{R_{2}^{5}}} - \frac{{30h{Z_0}{{\left( {{Z_0}+h} \right)}^3}}}{{R_{2}^{7}}}] \\ \end{gathered}$$

5 $$\begin{gathered} {R_1}=\sqrt {{{\left( {\sqrt {{x^2}+{y^2}} \sin \alpha - \lambda } \right)}^2}+{{\left( {\sqrt {{x^2}+{y^2}} \cos \alpha - \eta } \right)}^2}+{{\left( {z - h} \right)}^2}} \\ =\sqrt {\begin{array}{*{20}{c}} {{{\left( {\sqrt {{x^2}+{{\left( {r - \sqrt {{r^2} - {x^2}} } \right)}^2}} \sin \left( {\alpha - \beta } \right) - \lambda } \right)}^2}+} \\ {{{\left( {\sqrt {{x^2}+{{\left( {r - \sqrt {{r^2} - {x^2}} } \right)}^2}} \cos \left( {\alpha - \beta } \right) - \eta } \right)}^2}+{{\left( {z - h} \right)}^2}} \end{array}} \\ \end{gathered}$$

6 $$\begin{gathered} {R_2}=\sqrt {{{\left( {\sqrt {{x^2}+{y^2}} \sin \alpha - \lambda } \right)}^2}+{{\left( {\sqrt {{x^2}+{y^2}} \cos \alpha - \eta } \right)}^2}+{{\left( {z+h} \right)}^2}} \\ =\sqrt {\begin{array}{*{20}{c}} {{{\left( {\sqrt {{x^2}+{{\left( {r - \sqrt {{r^2} - {x^2}} } \right)}^2}} \sin \left( {\alpha - \beta } \right) - \lambda } \right)}^2}+} \\ {{{\left( {\sqrt {{x^2}+{{\left( {r - \sqrt {{r^2} - {x^2}} } \right)}^2}} \cos \left( {\alpha - \beta } \right) - \eta } \right)}^2}+{{\left( {z+h} \right)}^2}} \end{array}} \\ \end{gathered}$$

**Fig. 4:**
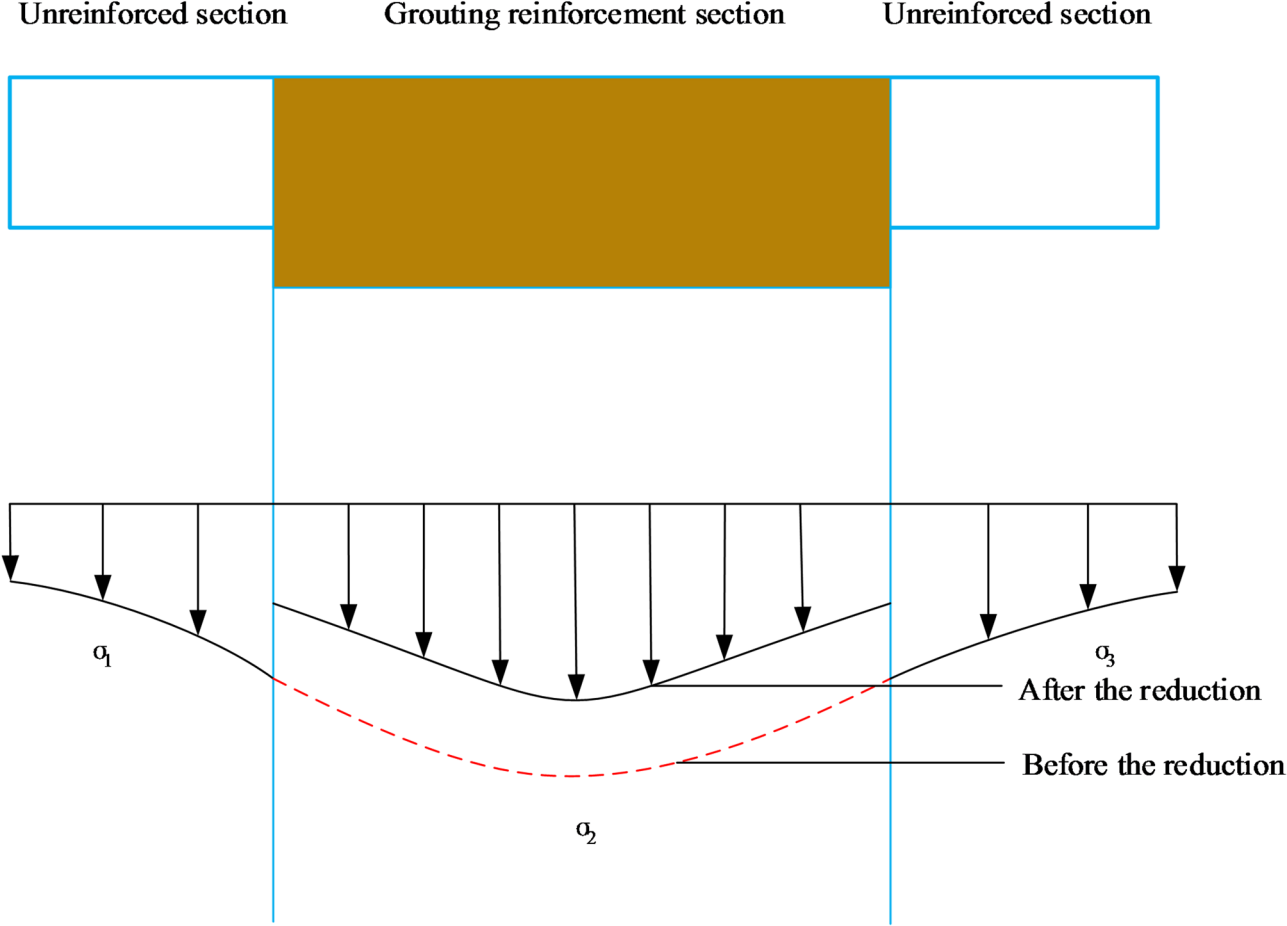
Stress reduction model.

Grouting reinforcement was performed near the centers of the new and old tunnels. As shown in Fig. 4, the additional stress on the surface of the unreinforced section on the left side of the existing tunnel is ${\sigma _1}$, the additional stress on the surface of the grout-reinforced section of the existing tunnel is ${\sigma _2}$, and the additional stress on the surface of the unreinforced section on the right side of the existing tunnel is ${\sigma _3}$.

The coordinate of the first point on the inner surface of the grouting ring is $\left( {x',y',z'} \right)$, and that on the outer surface of the grouting ring in the vertical direction is $\left( {x,y,z} \right)$. The relationship between ${\sigma _2}$ and ${\sigma _1}$(${\sigma _3}$) can be expressed as:

7 $${\sigma _2}=\frac{{{Q_2}}}{{{Q_1}}}{\sigma _3}=\frac{{{Q_2}}}{{{Q_1}}}{\sigma _1}$$

The stress reduction factor accounts for the difference in stress transfer between grouted and non-grouted zones. The stress reduction factors ${Q_1}$and ${Q_2}$ can be calculated as:

8a $$\begin{gathered} {Q_1}=\frac{1}{{8\pi \left( {1 - {\mu _1}} \right)}}[ - \frac{{\left( {1 - 2{\mu _1}} \right)\left( {z' - z} \right)}}{{R_{1}^{3}}}+\frac{{\left( {1 - 2{\mu _1}} \right)\left( {z' - z} \right)}}{{R_{2}^{3}}} - \frac{{3{{\left( {z' - z} \right)}^3}}}{{R_{1}^{5}}} \\ - \frac{{3\left( {3 - 4{\mu _1}} \right)z'{{\left( {z'+z} \right)}^2} - 3z\left( {z'+z} \right)\left( {5z' - z} \right)}}{{R_{2}^{5}}} - \frac{{30z'z{{\left( {z'+z} \right)}^3}}}{{R_{2}^{7}}}] \\ \end{gathered}$$

8b $$\begin{gathered} {Q_2}=\frac{1}{{8\pi \left( {1 - {\mu _2}} \right)}}[ - \frac{{\left( {1 - 2{\mu _2}} \right)\left( {z' - z} \right)}}{{R_{1}^{3}}}+\frac{{\left( {1 - 2{\mu _2}} \right)\left( {z' - z} \right)}}{{R_{2}^{3}}} - \frac{{3{{\left( {z' - z} \right)}^3}}}{{R_{1}^{5}}} \\ - \frac{{3\left( {3 - 4{\mu _2}} \right)z'{{\left( {z'+z} \right)}^2} - 3z\left( {z'+z} \right)\left( {5z' - z} \right)}}{{R_{2}^{5}}} - \frac{{30z'z{{\left( {z'+z} \right)}^3}}}{{R_{2}^{7}}}] \\ \end{gathered}$$

9 $$\begin{gathered} {R_1}=\sqrt {{{\left( {x - x'} \right)}^2}+{{\left( {y - y'} \right)}^2}+{{\left( {z - z'} \right)}^2}}, \hfill \\ {R_2}=\sqrt {{{\left( {x - x'} \right)}^2}+{{\left( {y - y'} \right)}^2}+{{\left( {z+z'} \right)}^2}} \hfill \\ \end{gathered}$$

where ${\mu _1}$ is the Poisson’s ratio of the soil, ${\mu _2}$ is the Poisson’s ratio of the grout-reinforced section. Therefore, $\sigma$ is calculated as follows based on Eq. (4):

10 $$\begin{gathered} {\sigma _2}\left( x \right)=\frac{{{Q_2}}}{{{Q_1}}}\mathop \smallint \nolimits_{{ - {R_0}}}^{{{R_0}}} \mathop \smallint \nolimits_{{ - {L_1}}}^{{{L_2}}} \frac{{qd\lambda d\eta }}{{8\pi \left( {1 - \mu } \right)}}[ - \frac{{\left( {1 - 2\mu } \right)\left( {{Z_0} - h} \right)}}{{R_{1}^{3}}}+\frac{{\left( {1 - 2\mu } \right)\left( {{Z_0} - h} \right)}}{{R_{2}^{3}}} \\ - \frac{{3{{\left( {{Z_0} - h} \right)}^3}}}{{R_{1}^{5}}} - \frac{{3\left( {3 - 4\mu } \right){Z_0}{{\left( {{Z_0}+h} \right)}^2} - 3h\left( {{Z_0}+h} \right)\left( {5{Z_0} - h} \right)}}{{R_{2}^{5}}} \\ - \frac{{30h{Z_0}{{\left( {{Z_0}+h} \right)}^3}}}{{R_{2}^{7}}}] \end{gathered} x \in \left[ {a,b} \right]$$

11 $$\begin{gathered} {\sigma _1}\left( x \right)={\sigma _3}\left( x \right)=\mathop \smallint \nolimits_{{ - {R_0}}}^{{{R_0}}} \mathop \smallint \nolimits_{{ - {L_1}}}^{{{L_2}}} \frac{{qd\lambda d\eta }}{{8\pi \left( {1 - \mu } \right)}}[ - \frac{{\left( {1 - 2\mu } \right)\left( {{Z_0} - h} \right)}}{{R_{1}^{3}}}+\frac{{\left( {1 - 2\mu } \right)\left( {{Z_0} - h} \right)}}{{R_{2}^{3}}} \\ - \frac{{3{{\left( {{Z_0} - h} \right)}^3}}}{{R_{1}^{5}}} - \frac{{3\left( {3 - 4\mu } \right){Z_0}{{\left( {{Z_0}+h} \right)}^2} - 3h\left( {{Z_0}+h} \right)\left( {5{Z_0} - h} \right)}}{{R_{2}^{5}}} \\ - \frac{{30h{Z_0}{{\left( {{Z_0}+h} \right)}^3}}}{{R_{2}^{7}}}] \\ x \in \left[ { - \infty ,a} \right]\bigcup {\left[ {b,\infty } \right]} \\ \end{gathered}$$

Equation (9) becomes

12 $$\begin{gathered} {R_1}=\sqrt {{{\left( {\sqrt {{x^2}+{y^2}} \sin \alpha - \lambda } \right)}^2}+{{\left( {\sqrt {{x^2}+{y^2}} \cos \alpha - \eta } \right)}^2}+{{\left( {z - h} \right)}^2}} \\ =\sqrt {\begin{array}{*{20}{c}} {{{\left( {\sqrt {{x^2}+{{\left( {r - \sqrt {{r^2} - {x^2}} } \right)}^2}} \sin \left( {\alpha - \beta } \right) - \lambda } \right)}^2}+} \\ {{{\left( {\sqrt {{x^2}+{{\left( {r - \sqrt {{r^2} - {x^2}} } \right)}^2}} \cos \left( {\alpha - \beta } \right) - \eta } \right)}^2}+{{\left( {z - h} \right)}^2}} \end{array}} \\ {R_2}=\sqrt {\begin{array}{*{20}{c}} {{{\left( {\sqrt {{x^2}+{y^2}} \sin \alpha - \lambda } \right)}^2}+{{\left( {\sqrt {{x^2}+{y^2}} \cos \alpha - \eta } \right)}^2}} \\ {+{{\left( {z+h} \right)}^2}} \end{array}} \\ =\sqrt {\begin{array}{*{20}{c}} {{{\left( {\sqrt {{x^2}+{{\left( {r - \sqrt {{r^2} - {x^2}} } \right)}^2}} \sin \left( {\alpha - \beta } \right) - \lambda } \right)}^2}+} \\ {{{\left( {\sqrt {{x^2}+{{\left( {r - \sqrt {{r^2} - {x^2}} } \right)}^2}} \cos \left( {\alpha - \beta } \right) - \eta } \right)}^2}+{{\left( {z+h} \right)}^2}} \end{array}} \\ \end{gathered}$$

### Finite difference method to solve the equation of the elastic foundation beam model

**Fig. 5:**
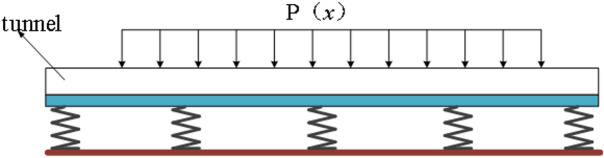
Pasternak foundation model.

As shown in Fig. 5, the Pasternak foundation model describes the shear and compressive properties of the soil. It has high accuracy and low computational complexity. Thus, this model was used to establish the differential equation to calculate the vertical deformation of the existing tunnel.

**Fig. 6:**
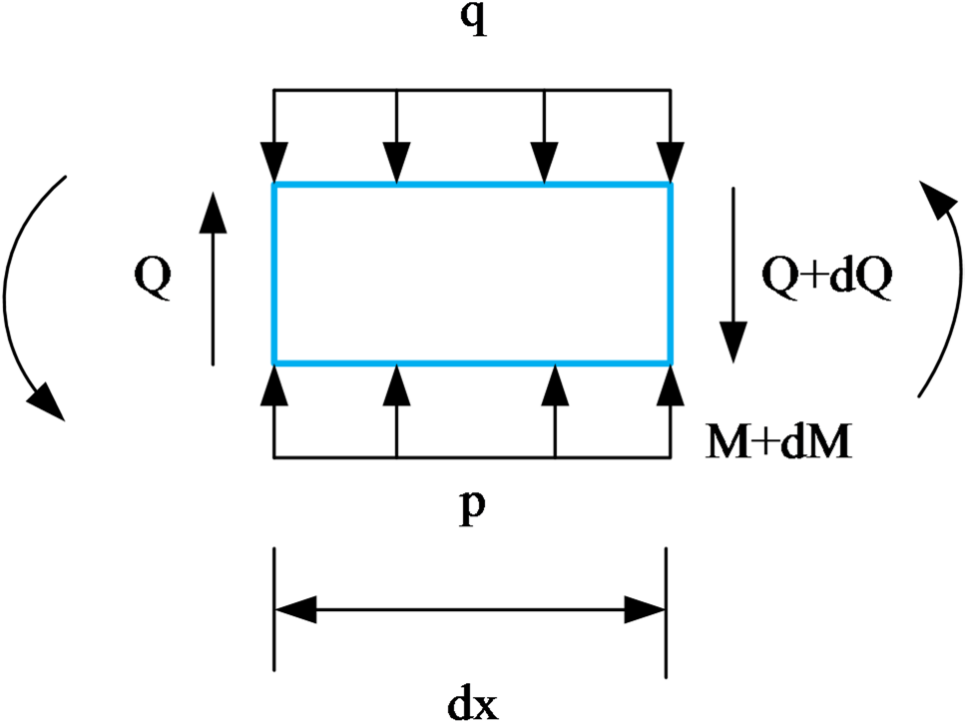
Microelement.

As shown in Fig. 6, a microelement with a length of *dx* is intercepted by the beam. The vertical force and moment equilibrium conditions are expressed as:

13 $$\sum {{F_y}} =0{\text{ }}Q - \left( {Q+dQ} \right)+kwdx - {q_{\left( x \right)}}dx=0$$

14 $$\sum {M=0} {\text{ }}M - \left( {M+dM} \right)+\left( {Q+dQ} \right)dx+{q_{\left( x \right)}}\frac{{{{\left( {dx} \right)}^2}}}{2} - kw\frac{{{{\left( {dx} \right)}^2}}}{2}=0$$

Combining Eqs. (13) and (14) and $\theta =\frac{{dw}}{{dx}}$ provides the following equation:

15 $${\left( {EI} \right)_{{\mathrm{eq}}}}\frac{{{d^4}w}}{{d{x^4}}}+kw={q_{\left( x \right)}}$$

Tanahashi^22^ used the Euler-Bernoulli beam model on the Pasternak foundation model to obtain:

16 $${\left( {EI} \right)_{{\mathrm{eq}}}}\frac{{{d^4}{w_{\left( x \right)}}}}{{d{x^4}}} - {G_{\mathrm{c}}}D\frac{{{d^2}{w_{\left( x \right)}}}}{{d{x^2}}}+kD{w_{\left( x \right)}}={q_{\left( x \right)}}D$$

where ${w_{\left( x \right)}}$ is the additional displacement of the existing tunnel caused by the new tunnel excavation, ${\left( {EI} \right)_{{\mathrm{eq}}}}$is the equivalent bending rigidity, ${G_c}$ is the shear parameter, *k* is the coefficient of the subgrade reaction, *D* is the outer diameter of the tunnel, ${q_{(x)}}$ is the additional stress caused by new tunnel construction on the existing curvilinear tunnel reinforced by circumferential grouting.

**Fig. 7:**
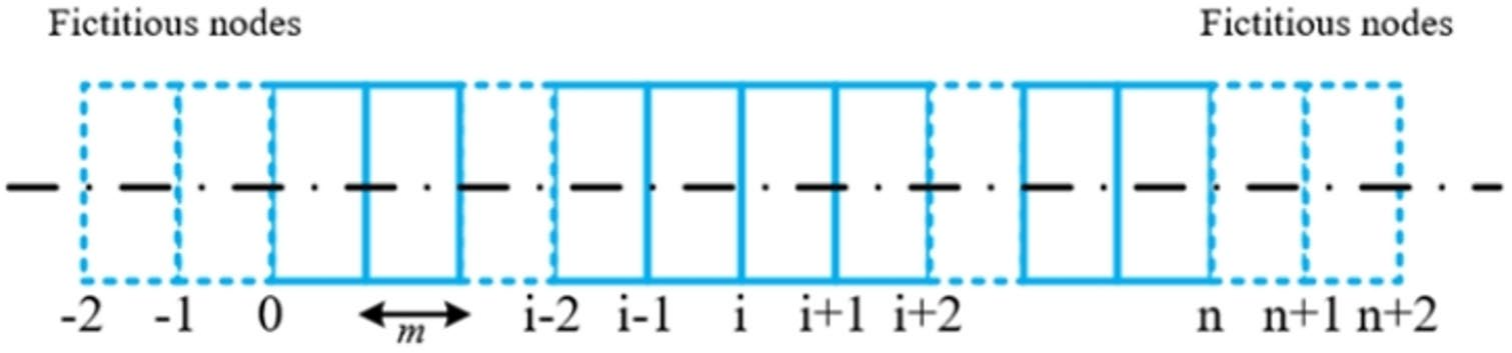
Discrete element analysis diagram of the existing tunnel.

The finite difference method is used to solve Eq. (16):

As shown in Figs. 4 and 7 virtual nodes are added to the existing tunnel to create *n* + 5 discrete units. If the unit length is *m*, the second- and fourth-order difference terms can be obtained as follows:

17 $$\frac{{{d^4}{w_{\left( x \right)}}}}{{d{x^4}}}=\frac{{6{w_i} - 4\left( {{w_{i+1}}+{w_{i - 1}}} \right)+\left( {{w_{i+2}}+{w_{i - 2}}} \right)}}{{{m^4}}}$$

18 $$\frac{{{d^2}{w_{\left( x \right)}}}}{{d{x^2}}}=\frac{{{w_{i+1}} - 2{w_i}+{w_{i - 1}}}}{{{m^2}}}$$

The finite difference expression of Eq. (16) is:

19 $$\begin{gathered} {\left( {EI} \right)_{{\mathrm{eq}}}}\frac{{6{w_i} - 4\left( {{w_{i+1}}+{w_{i - 1}}} \right)+\left( {{w_{i+2}}+{w_{i - 2}}} \right)}}{{{m^4}}} - {G_{\mathrm{c}}}D\frac{{{w_{i+1}} - 2{w_i}+{w_{i - 1}}}}{{{m^2}}} \hfill \\ {\text{ }}+kD{w_{\left( {{x_i}} \right)}}={q_{\left( x \right)}}D \hfill \\ \end{gathered}$$

If the tunnel is free at both ends at infinity, the boundary conditions and symmetry are expressed as:

20 $$\left\{ \begin{gathered} {M_n}={M_0}=0 \hfill \\ {Q_n}={Q_0}=0 \hfill \\ \end{gathered} \right.$$

This equation becomes:

21 $$\left\{ \begin{gathered} - {(EI)_{{\mathrm{eq}}}}\frac{{{w_{n+1}} - 2{w_n}+{w_{n - 1}}}}{{{m^2}}}= - {(EI)_{{\mathrm{eq}}}}\frac{{{w_1} - 2{w_0}+{w_{ - 1}}}}{{{m^2}}}=0 \hfill \\ - {(EI)_{{\mathrm{eq}}}}\frac{{{w_{n+2}} - 2{w_{n+1}}+2w_{{n - 1}}^{{}} - {w_{n - 2}}}}{{2{m^3}}}= - {(EI)_{{\mathrm{eq}}}}\frac{{{w_2} - 2{w_1}+2w_{{ - 1}}^{{}} - {w_{ - 2}}}}{{2{m^3}}}=0 \hfill \\ \end{gathered} \right.$$

22 $$\left\{ \begin{gathered} {w_{n+1}}=2{w_n} - {w_{n - 1}} \hfill \\ {w_{ - 1}}=2{w_0} - {w_1} \hfill \\ {w_{n+2}}=2{w_{n+1}} - 2{w_{n - 1}}+{w_{n - 2}} \hfill \\ {w_{ - 2}}={w_2} - 2{w_1}+2{w_{ - 1}} \hfill \\ {w_{n+2}}=4{w_n} - 4{w_{n - 1}}+{w_{n - 2}} \hfill \\ {w_{ - 2}}=4{w_0} - 4{w_1}+{w_2} \hfill \\ \end{gathered} \right.$$

Therefore, the deflection of the existing tunnel is defined as follows:

23 $$\left( {\left[ {{K_{\mathrm{T}}}} \right] - \left[ {{G_{\mathrm{c}}}} \right]+\left[ {{K_{\mathrm{s}}}} \right]} \right)\left\{ w \right\}=\left\{ Q \right\}$$

where $\left[ {{K_{\mathrm{s}}}} \right]=DkI$, and *I* is the identity matrix. The matrix displacement method is used; the matrices $\left[ {{K_{\mathrm{T}}}} \right]$ and $\left[ {{G_{\mathrm{c}}}} \right]$ are defined as:

24 $$\left[ {{K_{\mathrm{T}}}} \right]=\frac{{{{\left( {EI} \right)}_{{\mathrm{eq}}}}}}{{{m^4}}}{\left[ {\begin{array}{*{20}{c}} 2&{ - 4}&2&{}&{}&{}&{}&{}&0 \\ { - 2}&5&{ - 4}&1&{}&{}&{}&{}&{} \\ 1&{ - 4}&6&{ - 4}&1&{}&{}&{}&{} \\ {}&1&{ - 4}&6&{ - 4}&1&{}&{}&{} \\ {}&{}& \ddots & \ddots & \ddots & \ddots & \ddots &{}&{} \\ {}&{}&{}&1&{ - 4}&6&{ - 4}&1&{} \\ {}&{}&{}&{}&1&{ - 4}&6&{ - 4}&1 \\ {}&{}&{}&{}&{}&1&{ - 4}&5&{ - 2} \\ 0&{}&{}&{}&{}&{}&2&{ - 4}&2 \end{array}} \right]_{\left( {n+1} \right) \times \left( {n+1} \right)}}$$

25 $$\left[ {{G_{\mathrm{c}}}} \right]={G_{\mathrm{c}}}D{\left[ {\begin{array}{*{20}{c}} 0&0&0&{}&{}&{}&0 \\ 1&{ - 2}&1&{}&{}&{}&{} \\ {}&1&{ - 2}&1&{}&{}&{} \\ {}&{}& \ddots & \ddots & \ddots &{}&{} \\ {}&{}&{}&1&{ - 2}&1&{} \\ {}&{}&{}&{}&1&{ - 2}&1 \\ 0&{}&{}&{}&0&0&0 \end{array}} \right]_{\left( {n+1} \right) \times \left( {n+1} \right)}}$$

Let the invertible matrix $\left[ K \right]=\left[ {{K_{\mathrm{T}}}} \right] - \left[ {{G_{\mathrm{c}}}} \right]+\left[ {{K_{\mathrm{s}}}} \right]$; Eq. (23) becomes

26 $$\left\{ w \right\}={\left[ K \right]^{ - 1}}\left\{ Q \right\}$$

Equation (26) is the equation of the vertical deformation of the existing tunnel affected by the additional unloading stress due to new tunnel excavation. A case study was conducted to verify the theoretical equation.

## Case study

### Comparison of calculation results and monitoring data

**Fig. 8:**
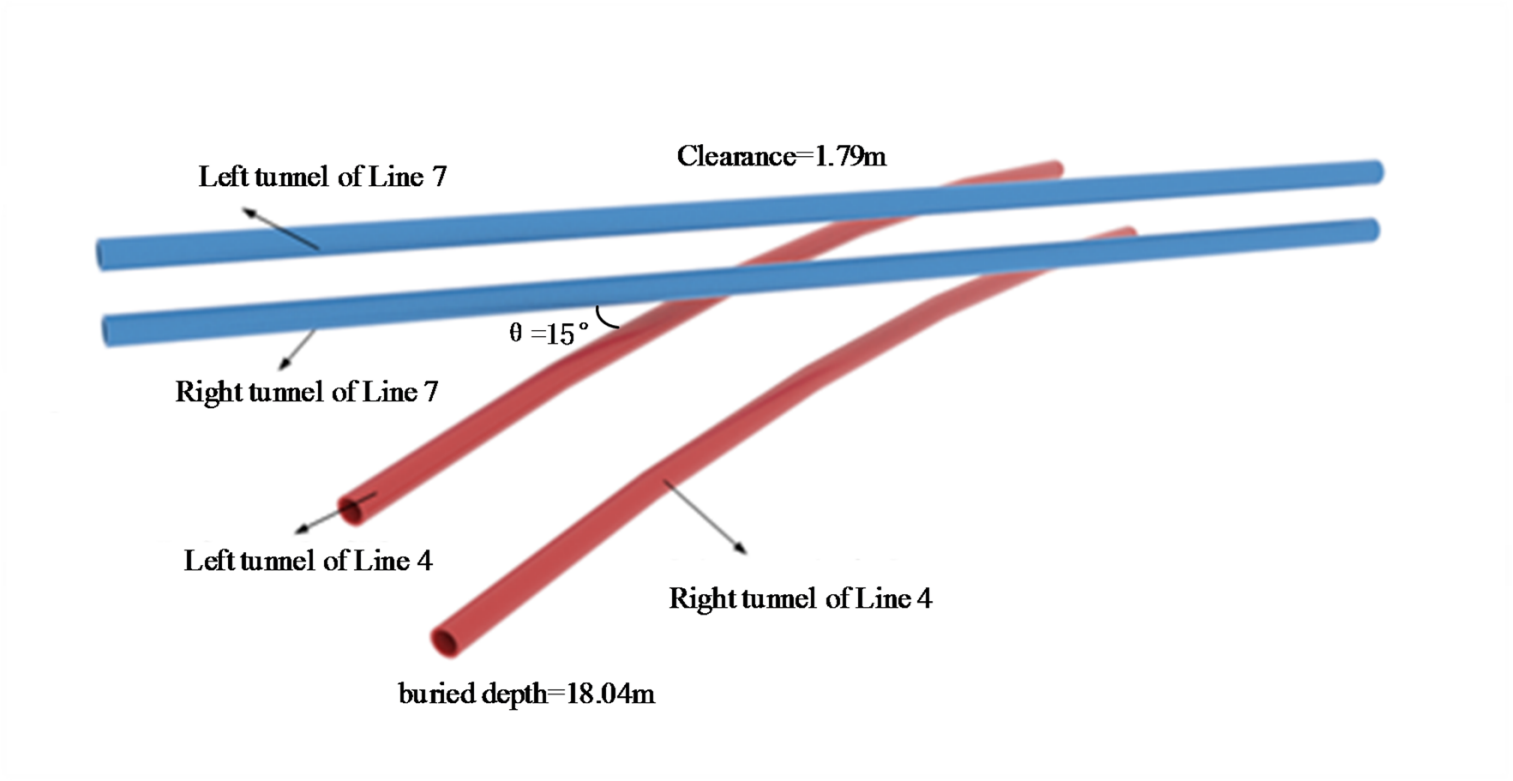
The position relationship between the Line 7 tunnel and the Line 4 tunnel.

The first phase of the Zhengzhou Metro Line 7 project from the Longmen Road Station to the Zhangjiacun Station in the Zhengzhou Metro Line 4 project used two earth pressure balance shield machines with a diameter of 6.46 m to perform the excavation. The outer diameter of the tunnel is 6.2 m, and the inner diameter is 5.5 m. The average buried depth of the axis of the newly built Line 7 tunnel is 7.30 m, and the average buried depth of the arch bottom is 10.05 m. The average buried depth of the left axis of the existing Line 4 is 15.29 m, and the average buried depth of the arch bottom is 18.04 m. The tunnel between Longmen Road Station and Zhangjiacun Station of Line 7 and the tunnel between Fengqing Road Station and Wenhua Road Station of Line 4 are shown in Fig. 8.

The flexural stiffness ($7.8 \times {10^4}MN \cdot {m^2}$) was calculated using the method of Shiba et al.^23^. The coefficient of the subgrade reaction of the foundation *k* was $5.41 \times {10^3}MN/{m^2}$^24^. We follow the methods of Tanahashi^22^ and Liang et al.^11^. to calculate the shear layer ${G_{\mathrm{c}}}$ ($45.59MN/m$). These parameters were derived from field geotechnical parameters obtained through case studies, ensuring the applicability and reliability of these critical input parameters in deformation analysis.

**Fig. 9:**
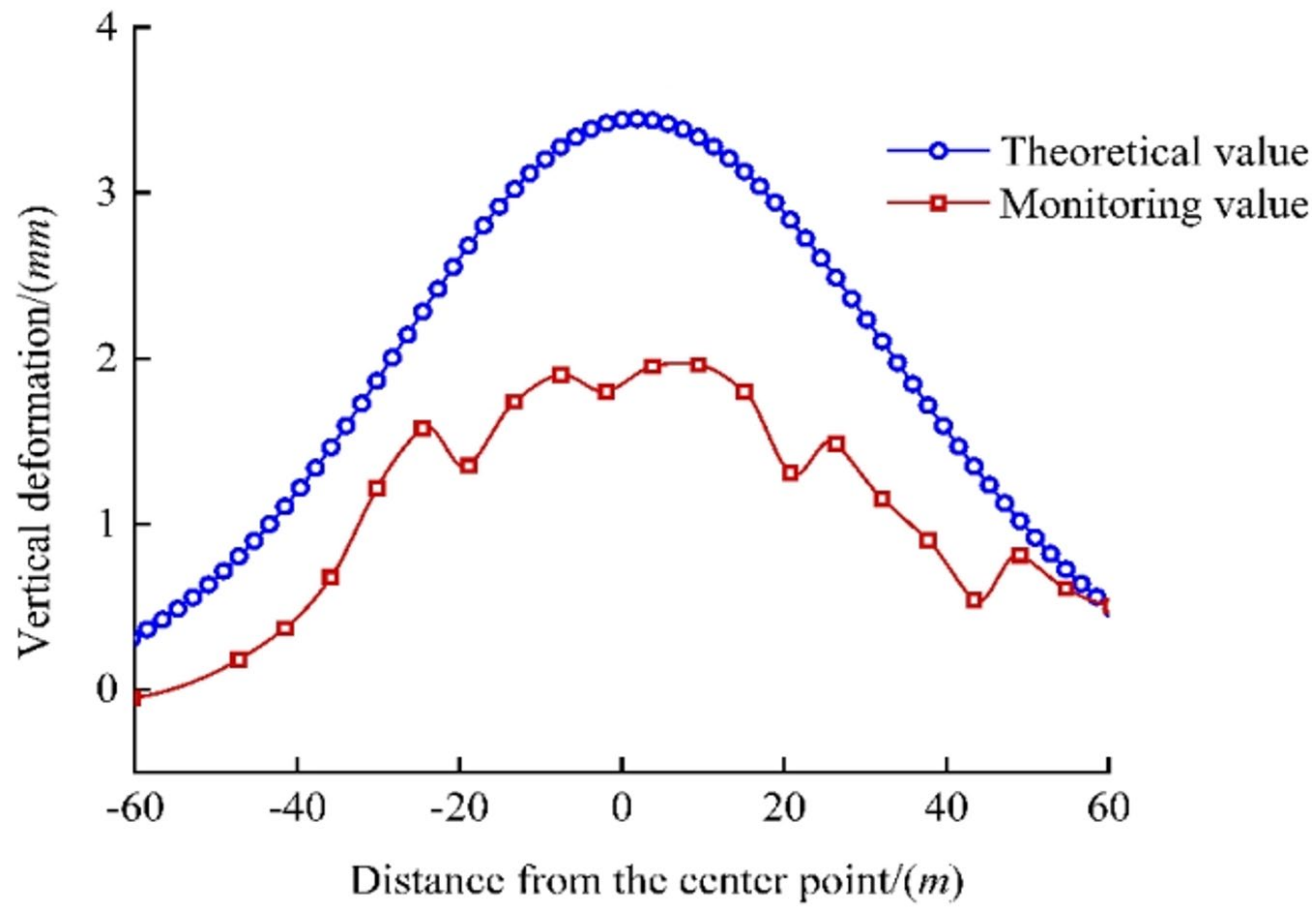
Comparison of vertical deformation of the existing tunnel’s left line by monitoring.

Combined with Eq. (26), the numerical solution to Eq. (15) was obtained using Eq. (26) and a Python program. The comparison with the monitoring values is shown in Fig. 9.

The maximum vertical displacement of the tunnel was 3.445 mm, exceeding the measured maximum displacement of 1.96 mm. The reason is that the calculated value is based on the arch roof, whereas the measured value is based on the arch bottom. The construction load of the new tunnel was transferred downward through the arch of the existing tunnel, and the arch bottom bore the load inside the tunnel. Therefore, the arch roof carried a larger load, and the deformation was larger. The theoretical model calculates deformation at the tunnel crown, while the monitoring data was obtained at the arch base. In shield tunnel engineering, it is well-established that the crown typically experiences larger displacements due to the direct transfer of overburden and excavation-induced stress. The predicted value (3.445 mm) serves as a conservative upper-bound estimate for the most critically loaded location. The agreement in the deformation trend and shape between the theoretical curve and field data, coupled with the fact that both values remain well within common safety thresholds for subway tunnel deformation (often on the order of 10–20 mm), confirms that the discrepancy is not only explainable but also acceptable from a practical engineering standpoint. The calculated and measured results are in good agreement, validating the proposed method.

## Analysis of factors influencing the deformation

### Influences of the clearance between existing and new tunnels

**Fig. 10:**
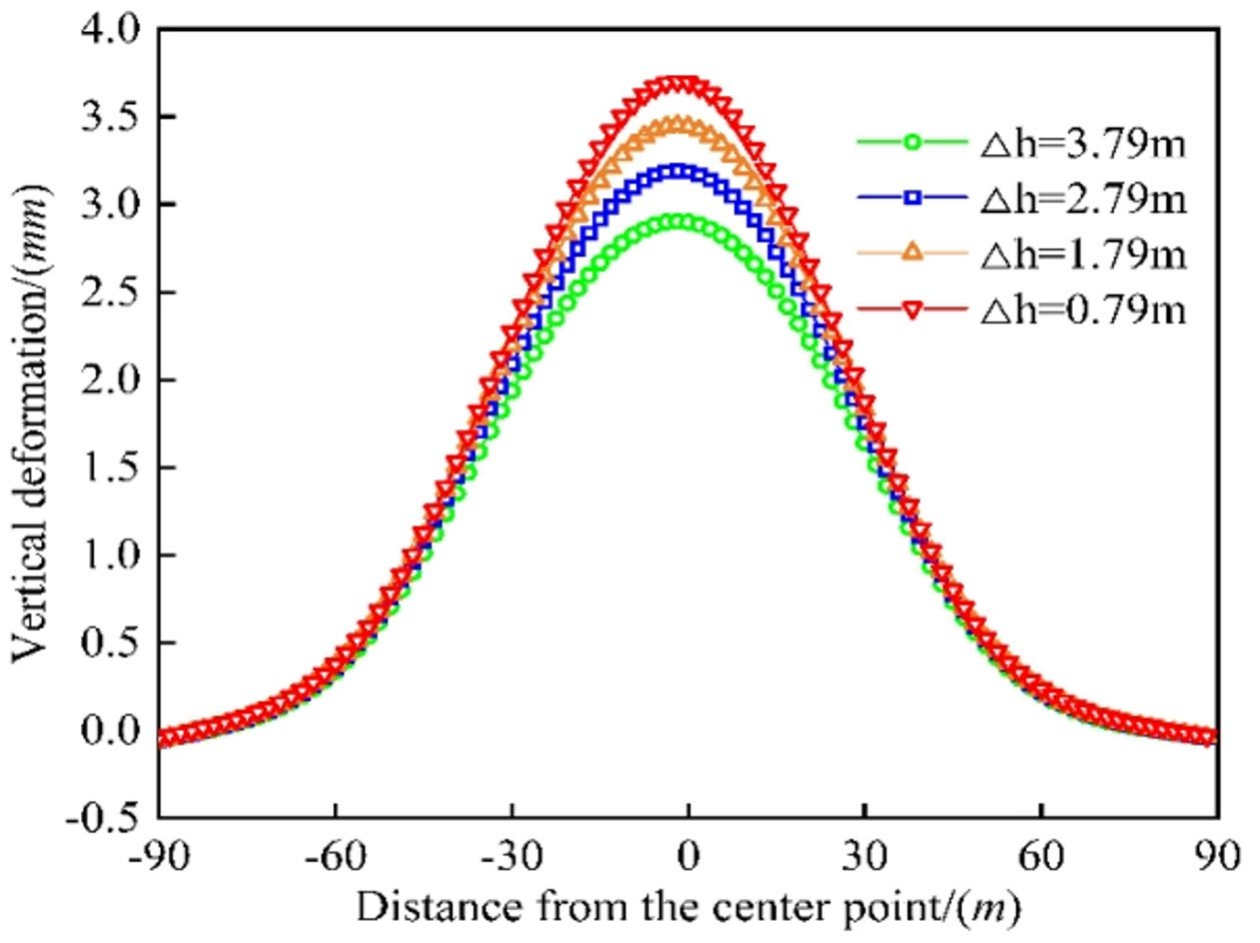
Comparison of existing tunnel deformation by different clearances.

As shown in Fig. 10, the maximum vertical deformation values of the existing tunnels were 3.699 mm, 3.445 mm, 3.190 mm, and 2.898 mm, respectively, for different distances between the old and new tunnels ($\Delta h$= 0.79 m, 1.79 m, 2.79 m, and 3.79 m). As the net distance between the old and new tunnels increased from 0.79 mm to 3.79 mm, the maximum vertical deformation of the existing tunnels decreased by 6.866%, 7.402%, and 9.153%. The reason is that the unloading stress decreased due to the excavation of the new tunnels, reducing the stress and deformation on the existing tunnels. The distance between the old and new tunnels should be as large as possible to reduce the deformation of the existing tunnels and reduce the construction risk.

### Influence of the angle between the two tunnels

**Fig. 11:**
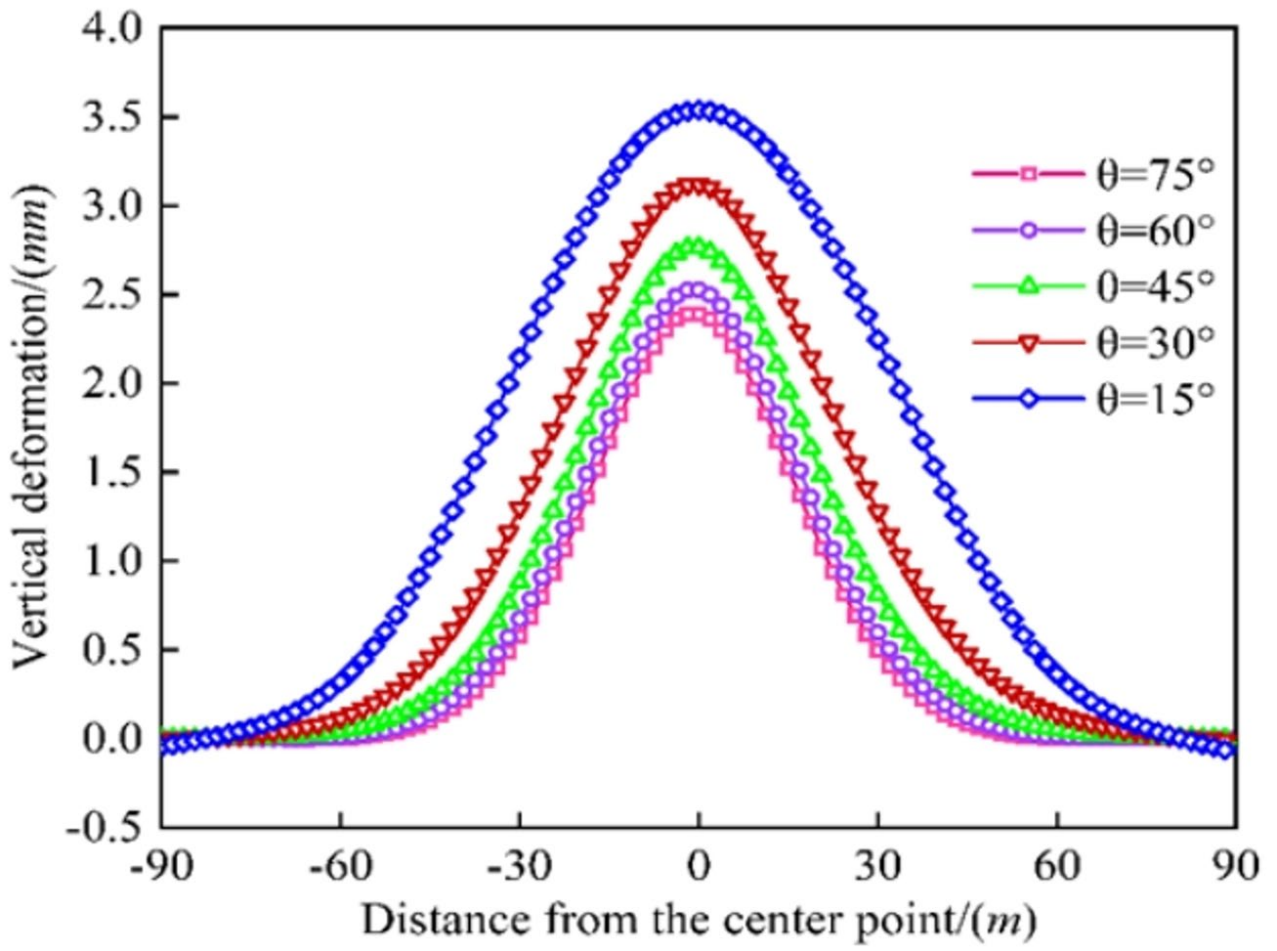
Comparison of existing tunnel deformation by different angles.

As shown in Fig. 11, the maximum vertical deformation of the existing tunnel as 3.537 mm, 3.118 mm, 2.765 mm, 2.536 mm, and 2.391 mm for an angle *θ* between the new and existing tunnels of 15°, 30°, 45°, 60°, and 75°, respectively. As the angle increased from 15° to 75°, the maximum vertical deformation of the existing tunnels decreased by 11.846%, 11.321%, 8.282%, and 5.717%. An increase in the angle reduced the earth pressure of the new tunnel on the existing tunnel, reducing the existing tunnel’s deformation. The angle between the new and existing tunnels should be as large as possible, especially for vertical and orthogonal crossings, to reduce the impact on the existing tunnel and ensure construction safety.

### Influence of the curvature radius of the existing tunnel

**Fig. 12:**
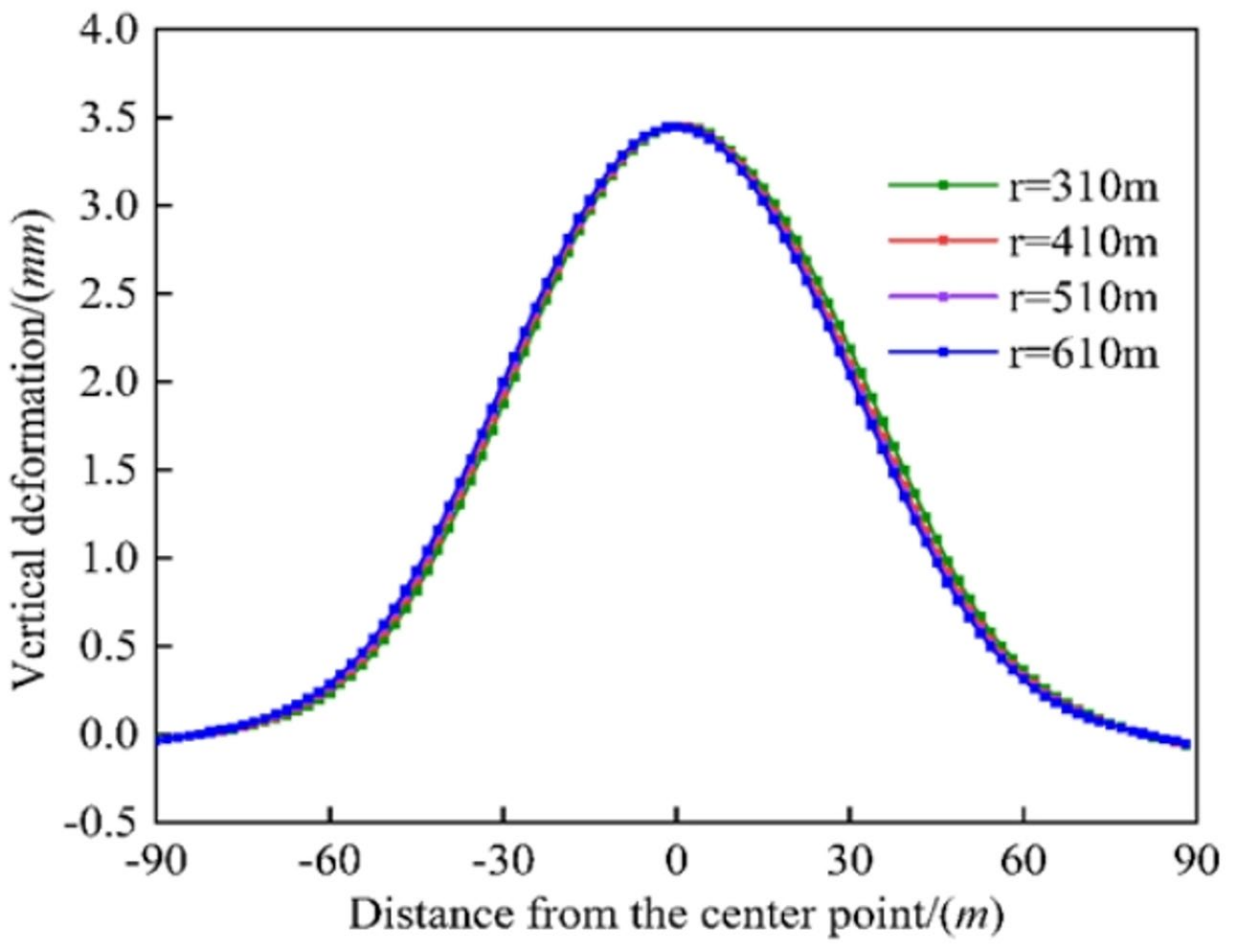
Comparison of existing tunnel deformation by different radius of curvature of the existing tunnel.

As shown in Fig. 12, as the curvature radius of the existing tunnel increased from 310 *m* to 610 *m*, the difference in the vertical deformation peak of the existing tunnel remained close to zero, indicating that the curvature radius had a negligible effect on the vertical deformation. Existing tunnels with different curvature radii are subject to the same additional stress at the center point, resulting in small differences in vertical displacement under different working conditions. However, the additional stress may differ further from the crossing point because it is less affected by the excavation disturbance. In general, the curvature radius of the existing tunnel has a negligible influence on the vertical deformation of the existing tunnel.

### Effect of the length of the grouting reinforcement of the existing tunnel

**Fig. 13:**
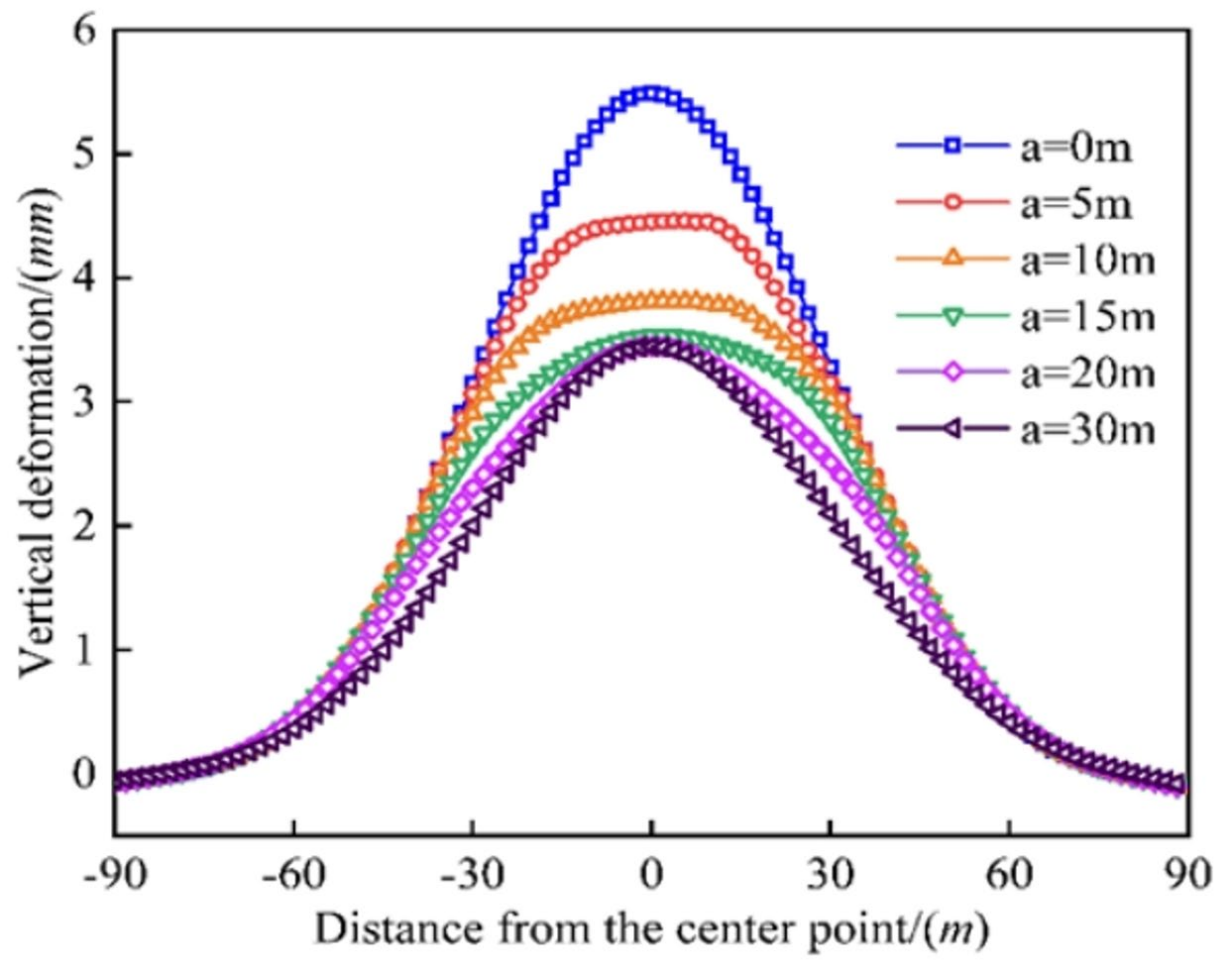
Comparison of existing tunnel deformation by different lengths of the grouting reinforcement.

As shown in Fig. 13, the maximum vertical deformation of the existing tunnel was 5.488 mm, 4.452 mm, 3.808 mm, 3.528 mm, 3.446 mm, and 3.445 mm when the length of the single-side grout-reinforced section was 0 m, 5 m, 10 m, 15 m, 20 m. and 30 m, respectively. As the length increased from 0 m to 30 m, the maximum vertical displacement of the existing tunnel decreased by 18.875%, 14.465%, 7.352%, 3.406%, and 0.029%. The vertical deformation and the deformation amplitude of the existing tunnel decreased with an increase in the length of the grout-reinforced section. For no grouting reinforcement, the curve of the additional stress had a normal distribution in the longitudinal direction of the existing tunnel. Since the elastic modulus was larger in the grout-reinforced section than in the surrounding soil layer, the additional stress was lower in the grout-reinforced section, and the stress mutation occurred at the edge of this section.

### Effect of Poisson’s ratio of the grout-reinforced soil on existing tunnels

**Fig. 14:**
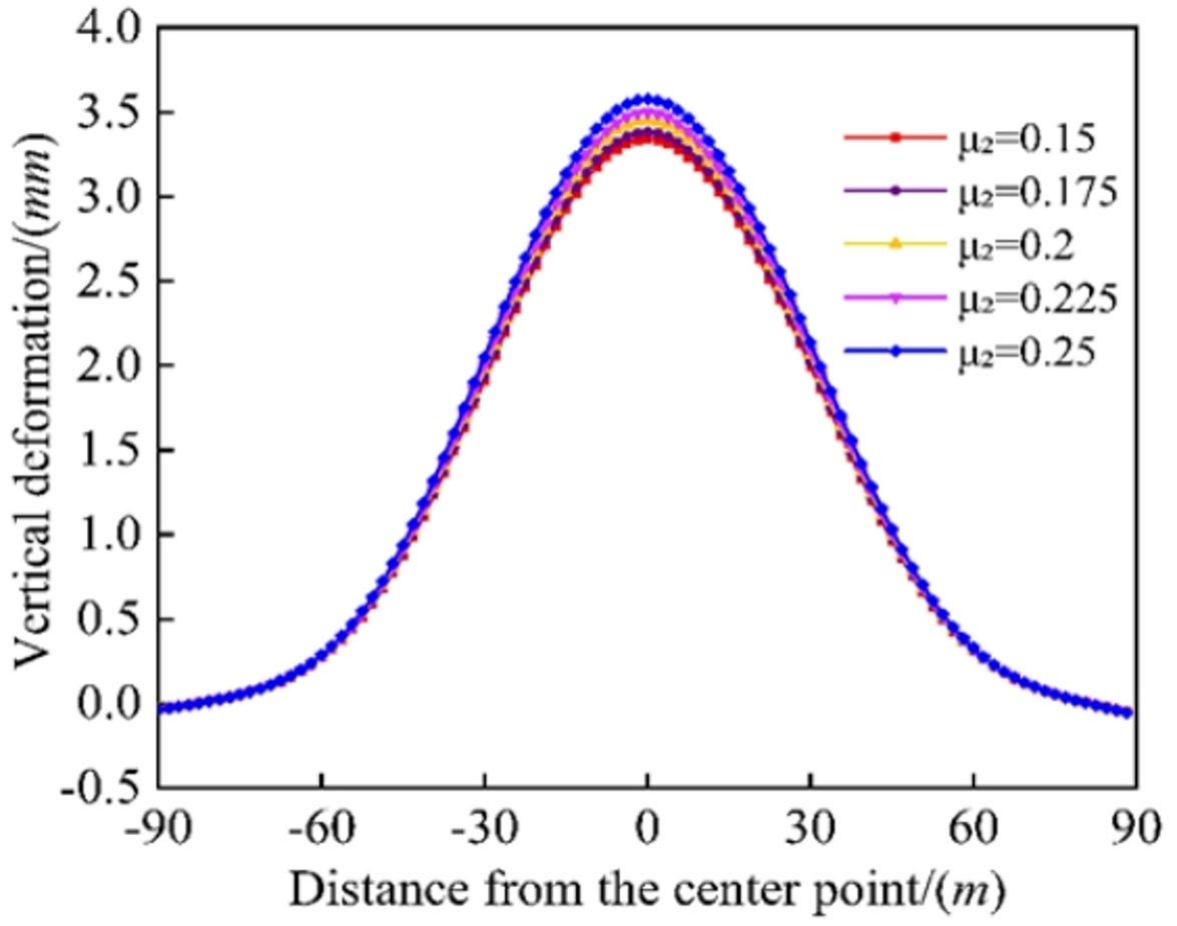
Comparison of existing tunnel deformation by different Poisson’s ratios of the grouting reinforcement.

As shown in Fig. 14, as the Poisson’s ratio of the grout-reinforced soil increased from 0.15 to 0.25, the maximum vertical deformation of the existing tunnel increased, but the rate of increase was relatively low. As the Poisson’s ratio increased, the soil in the grouted section became more compressed and stiffer and did not expand horizontally, increasing the additional stress exerted by the soil on the existing tunnel and the tunnel’s vertical deformation. However, due to the relatively short length of the grout-reinforced section and the limited interaction range between the grouted section and the soil around the existing tunnel, Poisson’s ratio had a negligible influence on the tunnel’s vertical deformation. Therefore, thme influence of the Poisson’s ratio depends on the length of the grouted section and the grouting material. Although Poisson’s ratio affected the vertical deformation of the existing tunnel, the influence was negligible.

## Conclusions

This study presents a refined two-stage method for predicting the deformation of existing curved subway tunnels induced by new shield tunnels crossing diagonally beneath. The method combines Mindlin’s solution for additional stress calculation with a Pasternak-foundation Euler–Bernoulli beam model, explicitly incorporating tunnel curvature and grouting reinforcement effects. Validation against monitoring data from the Zhengzhou Metro project demonstrates the model’s reliability and engineering applicability.

The analysis reveals that the clearance between the new and existing tunnels significantly influences deformation. As the distance decreases, the maximum vertical displacement of the existing tunnel increases considerably, suggesting that greater separation should be prioritized in design to reduce interaction effects and construction risk. As the curvature of the existing tunnel increased, the difference in the peak vertical deformation of the existing tunnels remained close to zero.

The crossing angle between tunnels is another critical parameter. The sensitivity of the vertical displacement of the existing tunnels to the angle between the old and new tunnels was relatively high. The new and existing tunnels should be orthogonal to minimize the deformation of the existing tunnel.

The length of the grout-reinforced section plays a vital role in controlling deformation. Increasing the grouting length reduces deformation amplitude, particularly in the initial extension range. However, the marginal benefit diminishes beyond a certain length, implying the existence of an economical and effective reinforcement design threshold.

Finally, the Poisson’s ratio of the grouted soil exhibits minimal impact on tunnel deformation, owing to the limited spatial extent of the reinforced zone. Thus, grouting material selection may focus on other mechanical properties rather than Poisson’s ratio.

This study provides a practical analytical tool; however, several inherent limitations stemming from its theoretical simplifications should be noted. The proposed two-stage method is grounded in linear elasticity and assumes the surrounding soil to be a homogeneous, isotropic medium. Consequently, it does not capture the time-dependent characteristics of soils, such as creep and consolidation, which may influence long-term displacements. Future refinements could involve integrating viscoelastic constitutive models and stepwise construction simulations to extend its applicability.

## Data Availability

All data or models that support the findings of this study are available from the corresponding author upon reasonable request.

## Declarations

### Date availability

All data or models that support the findings of this study are available from the corresponding author upon reasonable request.
